# Supplementary material for: Metabolic Dysfunction-Associated Steatotic Liver Disease Shapes a Distinct Semaphorin–Cytokine Immune Signature in Severe Community-Acquired Pneumonia
Source: Int J Mol Sci. 2025 Aug 21;26(16):8095. doi: 10.3390/ijms26168095 (PMC12386445; doi:10.3390/ijms26168095)

Supplementary Tables and Figures

Steatotic Liver Disease Shapes a Distinct Semaphorin–Cytokine Immune Signature in Severe Community-Acquired Pneumonia

Supplementary Table S1. Laboratory findings on admission

	MASLD	Non-MASLD	*p*-value
WBC, $\times 10^9$/L	13 (8,6-18)	8,3 (6,1-15)	**0,0118**
Neutrophils, %	85 (76-91)	84 (77-90)	0,8628
Lymphocytes, %	7,3 (4,5-14)	8,8 (5,9-14)	0,4126
Neutrophil/lymphocyte ratio	11 (5,7-20)	9,7 (5,8-15)	0,4653
Monocytes, %	6,7 (3,0-11,0)	5,5 (3,7-7,8)	0,2692
Hemoglobin, g/L	132 (119-143)	126 (115-141)	0,2599
Hematocrit, %	40 (37-43)	38 (35-42)	0,0640
MCV, fL	89 (86-94)	90 (83-93)	0,7960
Platelets, $\times 10^9$/L	201 (165-276)	205 (137-289)	0,4412
Platelet/lymphocyte ratio	265 (153-445)	277 (174-400)	0,8520
Sodium, mmol/L	139 (136-141)	137 (134-140)	0,1146
Potassium, mmol/L	4 (3,7-4,4)	3,9 (3,6-4,3)	0,4013
Glucose, mmol/L	7,1 (5,9-10)	6,7 (5,9-9,4)	0,6742
Urea, mmol/L	6,6 (4,7-11)	7,4 (5-12)	0,7306
Creatinine, µmol/L	97 (74-122)	82 (65-103)	0,0801
eGFR, mL/min/1.73 m^2	52 (40-70)	62 (39-84)	0,2794
Lactate, mmol/L	1,4 (0,93-3)	1,3 (1,1-1,7)	0,6858
Bilirubin, µmol/L	13 (9-20)	12 (8-15)	0,0770
AST, IU/L	55 (32-118)	48 (29-95)	0,4918
ALT, IU/L	45 (22-77)	34 (25-67)	0,2422
APRI score	0,71 (0,37-1,5)	0,71 (0,33-1,4)	0,8893
FIB-4 score	2,4 (1,1-4,6)	2,7 (1,5-4,6)	0,7526
GGT, IU/L	47 (34-91)	41 (20-86)	0,2160
ALP, IU/L	67 (52-103)	76 (50-109)	0,7724
LDH, IU/L	280 (224-396)	276 (213-398)	0,6561
CRP, mg/L	233 (144-376)	231 (124-339)	0,4040
Procalcitonin, µg/L	0,75 (0,26-3,2)	1,1 (0,23-3,4)	0,8762
hs-Troponin T, µg/L	0,028 (0,013-0,045)	0,019 (0,011-0,036)	0,1552
NT-pro-BNP, ng/L	1081 (265-2232)	729 (191-2823)	0,3689
Total proteins, g/L	61 (56-65)	60 (54-66)	0,4268
Albumin, g/L	31 (26-35)	29 (25-35)	0,7019
Total IgG, g/L	9,5 (7-12)	9,2 (7,5-13)	0,6447

Total IgA, g/L	2,3 (1,6-3,1)	2,3 (1,7-3,3)	0,8290
Total IgM, g/L	0,73 (0,51-1,2)	0,9 (0,57-1,4)	0,3866
**Lipid profile**			
Cholesterol, mmol/L	3,2 (2,7-4)	3,6 (2,6-4)	0,7491
Triglyceridi, mmol/L	1,9 (1,4-2,6)	1,8 (1,3-2,5)	0,9594
HDL, mmol/L	0,6 (0,5-0,8)	0,6 (0,5-0,78)	0,6934
LDL, mmol/L	1,9 (1,6-2,6)	2,1 (1,6-2,6)	0,4522
**Coagulation**			
PT, ratio	1,1 (0,94-1,2)	1,1 (1-1,3)	0,7434
INR	0,99 (0,9-1,1)	0,96 (0,91-1)	0,6402
Fibrinogen, g/L	7,4 (5,6-9,8)	6,8 (5,8-8,7)	0,6404
D-dimer, mg/L	1,7 (0,96-2,9)	2,5 (0,96-3,5)	0,2854
**Immunophenotyping of peripheral blood lymphocytes**			
CD3+ (T-lymphocytes), %	73 (61-79)	70 (62-76)	0,4226
CD19+ (B- lymphocytes), %	13 (7,4-25)	15 (9,6-22)	0,7355
CD4+ lymphocyte count/μL	553 (315-811)	477 (306-658)	0,2153
CD16/CD56 (NK-cells), %	9,8 (6,6-14)	12 (7,2-15)	0,2062
CD4+ (helper T-lymphocytes), %	49 (39-56)	47 (40-55)	0,4670
CD8+ (cytotoxic T-lymphocytes), %	19 (13-24)	21 (15-25)	0,3380
CD38+ (activated CD8+ lymphocytes), %	5,4 (3,3-9,4)	6,5 (3,9-12)	0,3580
HLA-DR+ (activated T- lymphocytes), %	9,1 (6,1-14)	8,7 (6,8-13)	0,8833
CD4+/CD8+ ratio	2,4 (1,9-3,5)	2,2 (1,7-3,1)	0,3663
**Serum protein electrophoresis**			
Alpha 1, %	5,8 (4,5-7,6)	6 (5-7,6)	0,3881
Alpha 2, %	18 (15-21)	18 (16-21)	0,9760
Beta, %	14 (13-15)	14 (13-16)	0,8870
Gamma, %	16 (13-19)	15 (13-18)	0,9986
Albumin/globulin ratio	0,84 (0,69-1)	0,83 (0,7-0,99)	0,8002

Data are presented as medians with interquartile ranges (IQR). Abbreviations: alkaline phosphatase (ALP); Alanine aminotransferase (ALT); Aspartate aminotransferase (AST); aspartate-aminotransferase-to-platelet ratio index (APRI); C-reactive protein (CRP); estimated glomerular filtration rate (eGFR); Fibrosis-4 index (FIB-4); gamma-glutamyl transferase (GGT); high-density lipoprotein cholesterol (HDL); international normalized ratio (INR); lactate dehydrogenase (LDH); low-density lipoprotein cholesterol (LDL); mean corpuscular volume (MCV); N-terminal pro-brain natriuretic peptide (NT-pro-BNP); natural killer lymphocyte subset (NK-cells); prothrombin time (PT); white blood cell count (WBC)

Supplementary Table S2. – Radiological findings on admission

	MASLD	Non-MASLD	*p*-value
**Radiologija**			
1 affected lung lobe	6 (11%)	4 (7%)	0,0720
2 affected lung lobe	12 (22%)	26 (48%)	
3 affected lung lobe	20 (37%)	11 (20%)	
4 affected lung lobe	10 (19%)	8 (15%)	
5 affected lung lobe	6 (11%)	5 (9%)	
Bilateral pneumonia	45 (83,3%)	42 (77,8%)	0,6276
Pleural effusion	27 (50,0%)	24 (44,4%)	0,7001

Supplementary Figure S1. Etiology of pneumonia

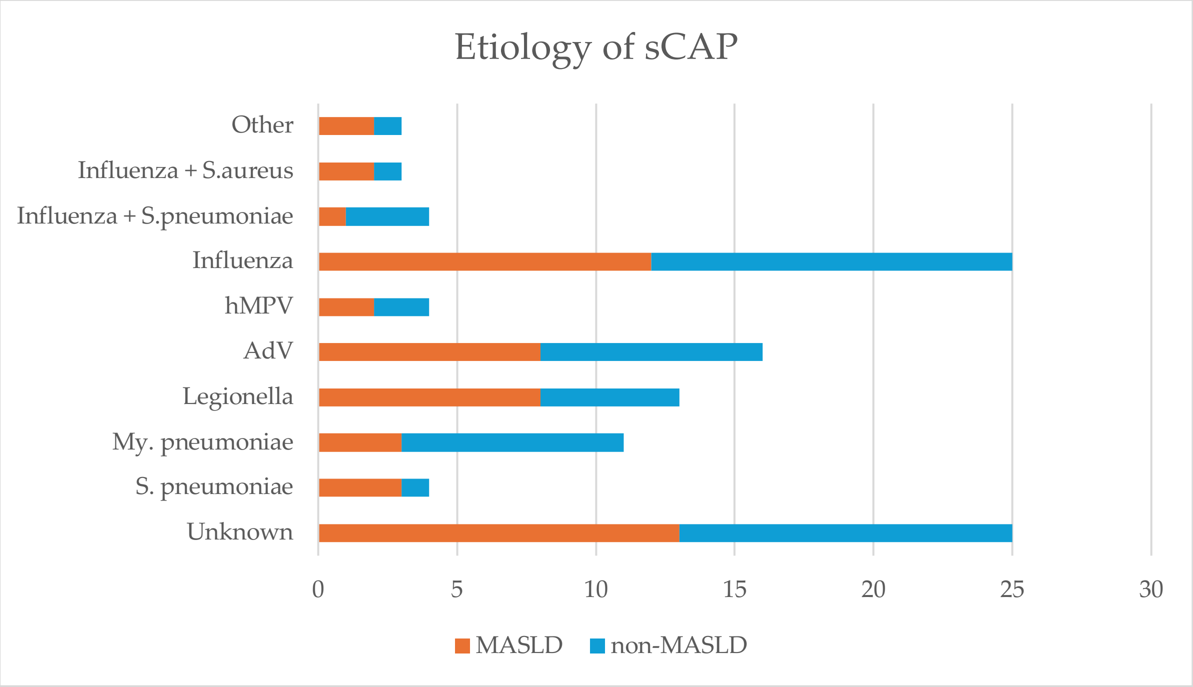

Abbreviations: adenovirus (AdV), human metapneumovirus (hMPV), *Mycoplasma pneumoniae* (*My. pneumoniae*), *Streptococcus pneumoniae* (*S. pneumoniae*), *Staphylococcus aureus* (*S. aureus*)

Supplementary Table S3. – Clinical presentation and treatment

	MASLD	Non-MASLD	*p*-value
Duration of symptoms at admission	6 (5-7)	7 (5-10)	0,0716
Catarrhal symptoms	25 (46,3%)	10 (18,5%)	**0,0020**
Dry cough	27 (50,0%)	27 (50,0%)	1,0000
Productive cough	25 (46,3%)	21 (38,9%)	0,4363
Gastrointestinal symptoms	16 (29,6%)	12 (22,2%)	0,3798
Max. body temperature, °C	39 (38,6-39,8)	39,2 (38,7-39,9)	0,7286
Antibiotic prior to admission	23 (42,6%)	20 (37%)	0,5554
β-lactam monotherapy	9 (16,67%)	10 (18,5%)	0,8005
Macrolide monotherapy	5 (9,3%)	3 (5,6%)	0,4624
Addition of a macrolide to a β-lactam	9 (16,7%)	7 (13%)	0,5880
Fluoroquinolone	0 (0%)	0 (0%)	1,0000
**Physical examination at study enrolment**			
Max. body temperature, °C	39 (38,6-39,8)	37,6 (36,9-38,3)	0,8712
Tachypnoea	54 (100,0%)	44 (81,5%)	**0,0009**
Dyspnoea	51 (94,4%)	48 (88,9%)	0,2963
Respiratory rate (min^{-1})	26 (24-29)	24 (22-30)	0,2004
Heart rate (min^{-1})	104 (89-120)	93 (80-108)	**0,0081**
FiO_2, %	40 (32-60)	40 (32-51)	0,9093
SpO_2, %	93 (90-95)	93 (90-95)	0,6725
SpO_2/FiO_2 ratio	242 (158-284)	241 (193-288)	0,6651
Systolic blood pressure, mmHg	123 (109-149)	120 (110-135)	0,4409
Diastolic blood pressure, mmHg	70 (64-80)	71 (66-79)	0,6868
Hypotension	12 (22,2%)	10 (18,5%)	0,6328
Shock	4 (7,4%)	1 (1,9%)	0,3632
Altered consciousness	9 (16,7%)	6 (11,1%)	0,4039
**Initial antimicrobial therapy at admission**			
β-lactam monotherapy	9 (16,7%)	7 (13%)	0,7873
β-lactam + macrolide/doxycycline	42 (77,8%)	45 (83,3%)	0,4658
Fluoroquinolone	1 (1,9%)	2 (3,7%)	1,0000
Oseltamivir monotherapy	2 (3,7%)	0 (0%)	0,4953
Oseltamivir (combined therapy)	24 (44,4%)	21 (38,9%)	0,5582
**Definitive antimicrobial treatment for sCAP during hospitalization**			
Subsequent modification of sCAP therapy	21 (38,9%)	21 (38,9%)	1,0000
Escalation of antimicrobial therapy	10 (18,5%)	4 (7,41%)	0,1502
De-escalation of antimicrobial therapy	11 (20,4%)	17 (31,5%)	0,1877
β-lactam monotherapy	9 (16,7%)	12 (22,2%)	0,4658
β-lactam + macrolide/doxycycline	27 (50,0%)	26 (48,1%)	0,8474
Macrolide monotherapy	8 (14,8%)	8 (14,8%)	1,0000
Fluoroquinolone	2 (3,7%)	4 (7,4%)	0,6785

Days of antimicrobial therapy	9 (5,3-10)	9 (7-11)	1,0000
Days of macrolide therapy	3 (3-5)	3 (3-5)	1,0000
**Immunomodulatory treatment**			
Corticosteroid therapy*	13 (24,1%)	16 (29,6%)	0,5148
Initial daily dose (methyl-prednisolone-equivalent), mg	80 (75-91,3)	80 (60-80)	1,0000
Duration of corticosteroid therapy, days	7 (3,5-10,5)	5 (5-6,8)	0,5403

Data are presented as medians with interquartile ranges (IQR) or frequencies (%). Abbreviations: fraction of inspired oxygen (FiO_2); peripheral capillary oxygen saturation measured by pulse oximetry (SpO_2)

* intravenous dexamethasone, hydrocortisone, or methyl-prednisolone

Supplementary Table S4. – Clinical course, outcomes and complications

**ICU supportive treatment**	**MASLD**	**Non-MASLD**	***p*-value**
CRRT	7 (13%)	3 (5,6%)	0,1842
HFNO	2 (3,7%)	5 (9,3%)	0,2410
NIV	3 (5,6%)	2 (3,7%)	0,6470
IMV	15 (27,8%)	6 (11,1%)	**0,0287**
vvECMO	6 (11,1%)	2 (3,7%)	0,1418
Vasopressors	13 (24.1%)	4 (7.4%)	**0,0323**

Abbreviations: continuous renal replacement therapy (CRRT); high-flow nasal oxygen HFNO); invasive mechanical ventilation (IMV); non-invasive mechanical ventilation (NIV); veno-venous extracorporeal membrane oxygenation (vvECMO)

	**MASLD**	**Non-MASLD**	***p*-value**
**Renal dysfunction***	13 (24,1%)	13 (24,1%)	1,0000
KDIGO stage	2 (1-3)	1 (1-3)	0,5421
Oliguria	2 (3,7%)	2 (3,7 %)	1,0000
Anuria	4 (7%)	2 (3,7 %)	0,4008
**Cardiovascular complications (episodes)**	15 (27,8%)	7 (13,0%)	0,0930
Pulmonary embolism/thrombosis	4 (7,4%)	2 (3,7%)	0,4008
Atrial fibrillation	2 (3,7%)	2 (3,7%)	1,0000
Myocarditis	1 (1,9%)	1 (1,9%)	1,0000
Heart failure*	6 (11,1%)	2 (3,7%)	0,1416
Cardiac arrest with successful resuscitation	2 (3,7%)	0 (0%)	0,1534
**Nosocomial infections (episodes)**	14 (25,9)	7 (13,0%)	0,1435
Urinary tract infection	4 (7,4%)	6 (11,1%)	0,7417
Clostridioides enterocolitis	1 (1,9%)	0 (0%)	1,0000
Pneumonia (non-VAP)	0 (0%)	0 (0%)	1,0000
VAP	5 (9,3%)	1 (1,9%)	0,2055
Sepsis	3 (5,6%)	0 (0%)	0,2430
**Other**			
Pleural empyema	0 (0%)	1 (1,9%)	0,3151
Pneumothorax	0 (0%)	2 (3,7%)	0,1534

Data are presented as medians with interquartile ranges (IQR) or frequencies (%). Abbreviations: "Kidney Disease: Improving Global Outcomes" criteria (KDIGO); ventilator-associated pneumonia (VAP)
* new-onset or acute exacerbation of a pre-existing chronic disease

Supplementary Figure S2 – Association of FAST score with sCAP clinical outcomes

Receiver operating characteristic (ROC) curves showing the predictive performance of the FAST score at admission for IMV, CRRT and mortality.

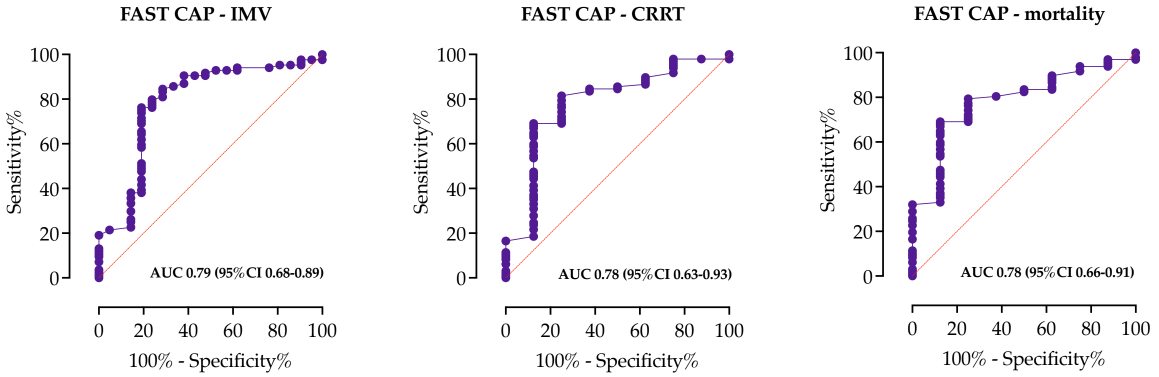

Association between FAST score and time to clinical stability.
(A) Simple linear regression showing a significant positive association between FAST score at admission and time to clinical stability. (B) Kaplan–Meier survival curve demonstrating the probability of achieving clinical stability by Day 7 of hospitalization, stratified by FAST score

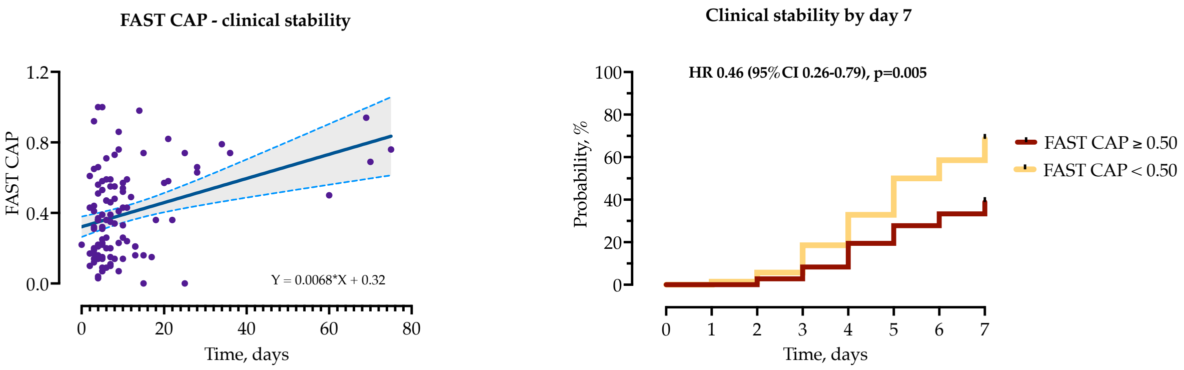

Supplementary Table S5. – Univariable and multivariable analysis for identification of factors associated with the need for invasive mechanical ventilation.

	**Univariable analysis**		**Multivariable analysis***	
	OR (95% CI)	p-value	OR (95% CI)	p-value
Male sex	1,1 (0,39-3,3)	0,9056		
**Age >60 years**	1,4 (1,0-2,2)	0,0124	6,3 (1,8-30)	0,0087
Type 2 diabetes mellitus	0,59 (0,13-2,0)	0,4188		
Arterial hypertension	0,67 (0,26-1,8)	0,4187		
Dyslipidemia	0,50 (0,13-1,5)	0,2212		
BMI >30 kg/m^2	0,90 (0,28-2,6)	0,8451		
**MASLD**	3,1 (1,1-9,3)	0,0266	3,8 (1,1-15)	0,0427
**FAST score >0,5**	10 (3,5-32)	<0,0001	7,8 (11-73)	0,0463
Tobacco or e-cigarette smoking	3,4 (1,2-11)	0,0261		
Influenza	1,1 (0,34-2,9)	0,9281		
Legionellosis	0,73 (0,11-3,0)	0,6859		
SOFA score >5	13 (4,3-39)	<0,0001		
PSI >120	4,5 (1,7-12)	0,0031		
**SMART-COP >6**	42 (12-182)	<0,0001	79 (12-1034)	<0,0001

* logistic regression analysis model AUC 0,94 (95% CI 0,88-0,99), p<0,0001

Supplementary Table S6. – Serum cytokine concentrations on Day 1 and Day 5, stratified by MASLD status.

	MASLD			Non-MASLD			MASLD *vs.* non-MASLD<br>p-value / difference (95% CI)	
	Day 1	Day 5	*p-value*<br>difference (95% CI)	Day 1	Day 5	*p-value*<br>difference (95% CI)	Day 1	Day 5
IL-1β	29,1<br>(18,9-39,8)	21,7<br>(18,0-40,7)	*0,4194*<br>3,1 (-4,5 – 11)	29,2<br>(13,3-52,0)	24,9<br>(19,1-46,6)	*0,1370*<br>5,7 (-1.9 – 13)	*0,7071*<br>-3,2 (-20 – 14)	*0,9411*<br>-0,64 (-18 – 16)
IL-2	6,3<br>(2,8-9,6)	3,9<br>(1,4-5,6)	***0,0120***<br>1,9 (0,43 - 3,4)	3,7<br>(2,2-5,9)	4,5<br>(2,8-7,7)	*0,1615*<br>-1,1 (-2,6 - 0,45)	***0,0434***<br>2,7 (0,08 - 5,2)	*0,7972*<br>-0,34 (-2,9 – 2,2)
IL-4	3,0<br>(1,2-12,4)	2,6<br>(1,2-9,9)	*0,3383*<br>3,4 (-3,6 – 10)	2,6<br>(1,2-11,2)	2,5<br>(1,2-11,5)	*0,7702*<br>-0,97 (-7,5 - 5,6)	*0,4538*<br>4,9 (-7,9 – 18)	*0,9375*<br>0,51 (-12 – 13)
IL-6	137,7<br>(71,7-425,2)	28,7<br>(10,0-91,2)	***0,0208***<br>821 (128 – 1514)	120,7<br>(33,6-350,7)	37,6<br>(7,5-92,8)	***0,0396***<br>729 (35 – 1422)	*0,6206*<br>190 (-565 – 944)	*0,7991*<br>98 (-657 – 852)
IL-8	21,3<br>(13,1-40,7)	12,9<br>(10,7-22,9)	0,1299<br>10 (-3,1 – 24)	20,3<br>(11,07-33,4)	15,9<br>(10,7-23,8)	*0,2403*<br>8,0 (-5,5 – 22)	*0,3007*<br>7,2 (-6,5 – 21)	*0,4854*<br>4,9 (-8,8 – 19)
IL-10	18,7<br>(8,7-39,4)	10,3<br>(5,5-20,9)	***0,0003***<br>15 (6,8 – 23)	10,0<br>(6,1-27,0)	8,0<br>(4,3-12,7)	***0,0118***<br>10 (2,3 – 18)	***0,0457***<br>9,4 (0,18 – 19)	*0,2987*<br>4,9 (-4,3 – 14)
IL-12p70	7,2<br>(2,1-18,8)	5,2<br>(2,1-15,2)	*0,5328*<br>1,0 (-2,2 - 4,3)	6,4<br>(2,1-11,5)	7,1<br>(2,2-13,3)	*0,7212*<br>0,58 (-2,7 – 3,8)	*0,2547*<br>4,1 (-3,0 – 11)	*0,3092*<br>3,6 (-3,4 – 11)
IL-17A	12,4<br>(7,6-25,2)	8,7<br>(3,1-18,8)	***0,0031***<br>5,8 (2,0 - 9,5)	6,0<br>(2,4-17,2)	8,1<br>(3,1-17,5)	*0,8689*<br>0,30 (-3,3 – 3,9)	***0,0345***<br>6,0 (0,44 – 12)	*0,8461*<br>0,55 (-5,0 - 6,1)
TNF-α	3,8<br>(1,9-9,9)	3,3<br>(1,9-7,9)	*0,4248*<br>0,90 (-1,3 - 3,1)	3,6<br>(1,7-8,4)	4,9<br>(2,0-10,0)	*0,3815*<br>-0,99 (-3,2 – 1,3)	*0,9322*<br>0,17 (-3,9 – 4,2)	*0,4012*<br>-1,7 (-5,7 – 2,3)
IFN-γ	17,5<br>(5,8-45,7)	5,5<br>(2,9-15,5)	***0,0277***<br>24 (2,6 – 45)	14,0<br>(5,6-46,7)	10,8<br>(5,4-28,4)	*0,0724*<br>19 (-1,8 – 41)	*0,4955*<br>-8,2 (-32 – 16)	*0,3020*<br>-12 (-36 – 11)
MCP-1<br>(CCL2)	384,8<br>(216,5-593,4)	269,4<br>(157,2-406,3)	*0,1227*<br>506 (-139 – 1150)	291,5<br>(162,2-771,7)	240,4<br>(156,3-349,6)	***0,0282***<br>723 (79 – 1367)	*0,5781*<br>-188 (-852 – 477)	*0,9303*<br>30 (-635 – 694)
CXCL10	3130<br>(1338-5452)	798<br>(493-1587)	***<0,0001***<br>2805 (1597 – 4013)	1151<br>(450-4494)	430<br>(308-876)	***0,0023***<br>1903 (695 – 3111)	***0,0153***<br>1623 (315 – 2931)	*0,2784*<br>721 (-587 – 2029)
TGF-β1	196,2<br>(115,3-298,4)	248,9<br>(125,1-476,2)	*0,0885*<br>-69 (-149 – 11)	100,1<br>(67,3-225,4)	176,0<br>(79,1-364,6)	***0,0119***<br>-91 (-161 - -21)	***0,0236***<br>107 (14 – 199)	*0,0702*<br>85 (-7,1 – 177)

Supplementary Table S7. – Serum semaphorin concentrations on Day 1 and Day 5, stratified by MASLD status.

	MASLD			Non-MASLD			MASLD *vs.* non-MASLD<br>p-value difference (95% CI)	
SEMA	Day 1	Day 5	*p-value*<br>difference (95% CI)	Day 1	Day 5	*p-value*<br>difference (95% CI)	Day 1	Day 5
3A	16 (14-17)	14 (13-17)	***0,0042***<br>1,63<br>(0.53- 2.72)	15 (13-16)	16 (14-17)	***0,0039***<br>-1,14<br>(-2.2-0.09)	***0,0412***<br>1,55<br>(0,06-3,04)	*0,1493*<br>-1,09<br>(-2.58- 0.39)
3F	8,9 (7,8-11)	8,8 (7,5-11)	0,9608<br>-0,025<br>(-1.1 - 1.0)	9,1 (7,8-11)	9,1 (7,5-11)	*0,3545*<br>-0,47<br>(-1.5 - 0.53)	*0,5175*<br>0,41<br>(-0.84-1.66)	*0,9577*<br>-0,03<br>(-1.3-1.2)
4D	39 (26-57)	44 (24-65)	*0,3493*<br>-2,9<br>(-9.1 - 3.2)	29 (21-46)	38 (19-64)	***0,0314***<br>-6,6<br>(-13 - -0.60)	*0,1807*<br>7,1<br>(-3.4 – 18)	*0,5159*<br>3,5<br>(-7.0 – 14)
5A	15 (3,7-26)	11 (3,9-26)	*0,9783*<br>0,08<br>(-5.9 - 6.0)	24 (8,3-49)	18 (6,8-33)	*0,0688*<br>5,7<br>(-0.45 – 12)	***0,0125***<br>-15<br>(-26 - -3.2)	*0,1203*<br>-9,1<br>(-21 - 2.4)
7A	31 (24-41)	25 (19-35)	***<0,0001***<br>6,7<br>(3.7 - 9.7)	28 (23-32)	23 (18-30)	***0,0299***<br>3,3<br>(0.33 - 6.3)	***0,0430***<br>4,6<br>(0.14 - 9.0)	*0,5955*<br>1,2<br>(-3.2 - 5.6)

Supplementary Figure S3 – Paired serum and BALF analysis of semaphorins concentrations.

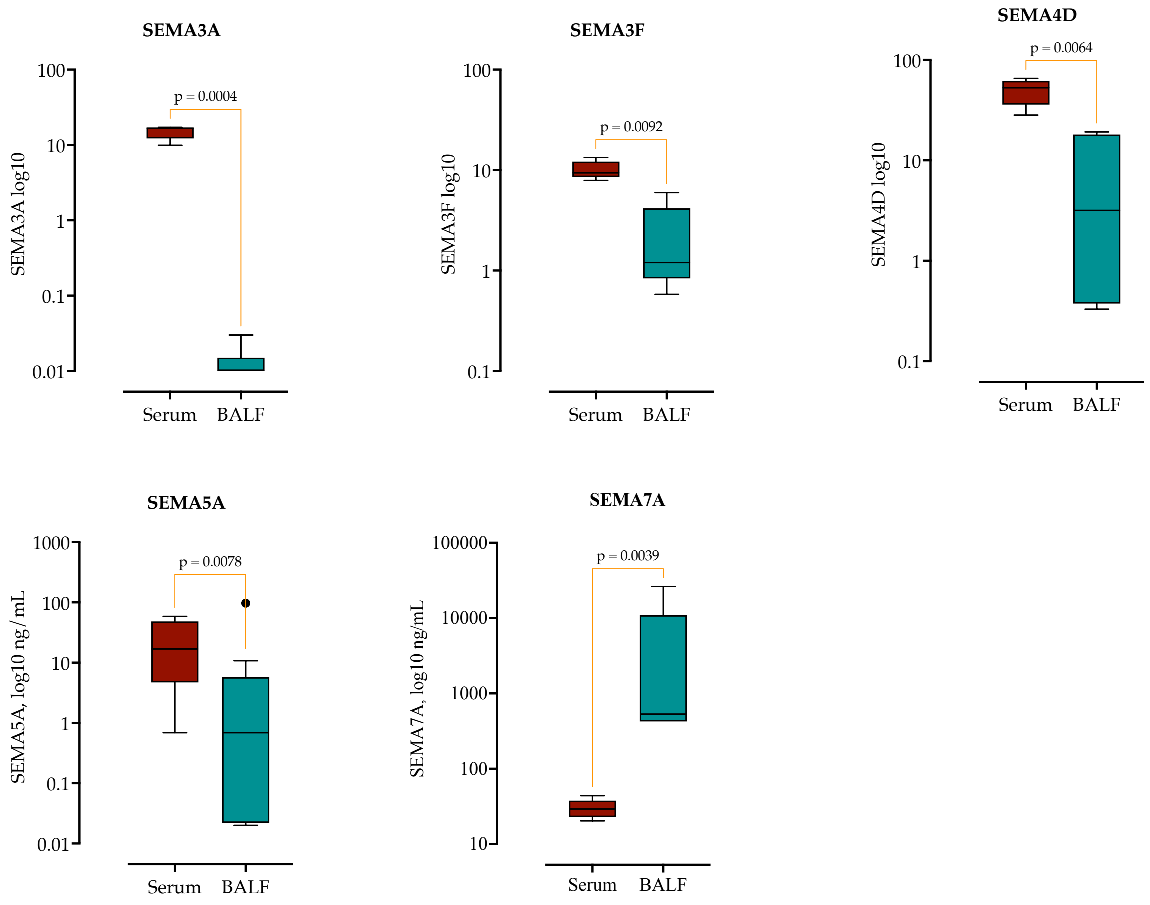

Supplementary Figure S4 – Heatmap showing correlation analysis between semaphorins and cytokines in BALF

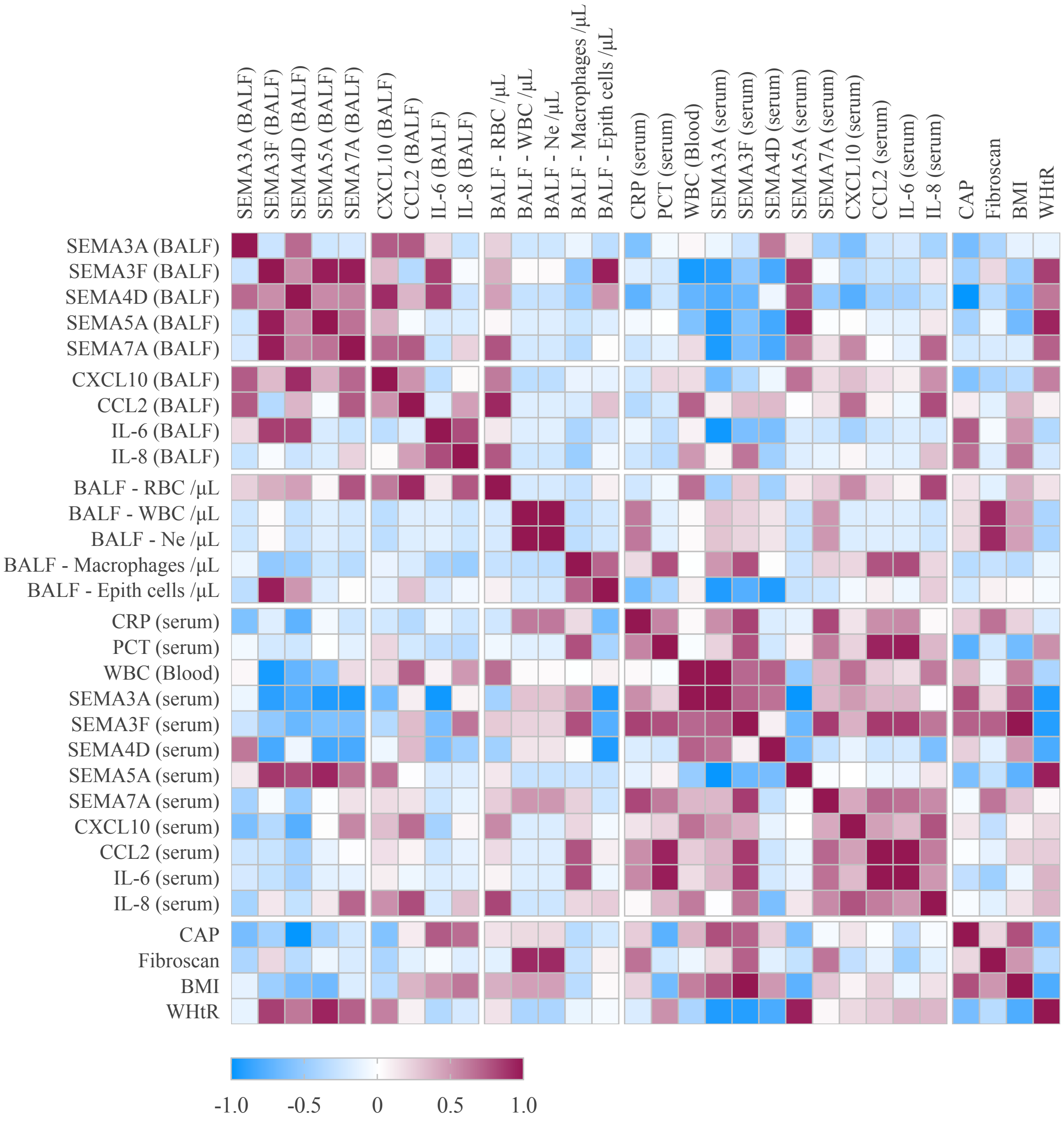

Supplementary Figure S5 – Heatmap showing correlation analysis between semaphorins and T-cell subtypes

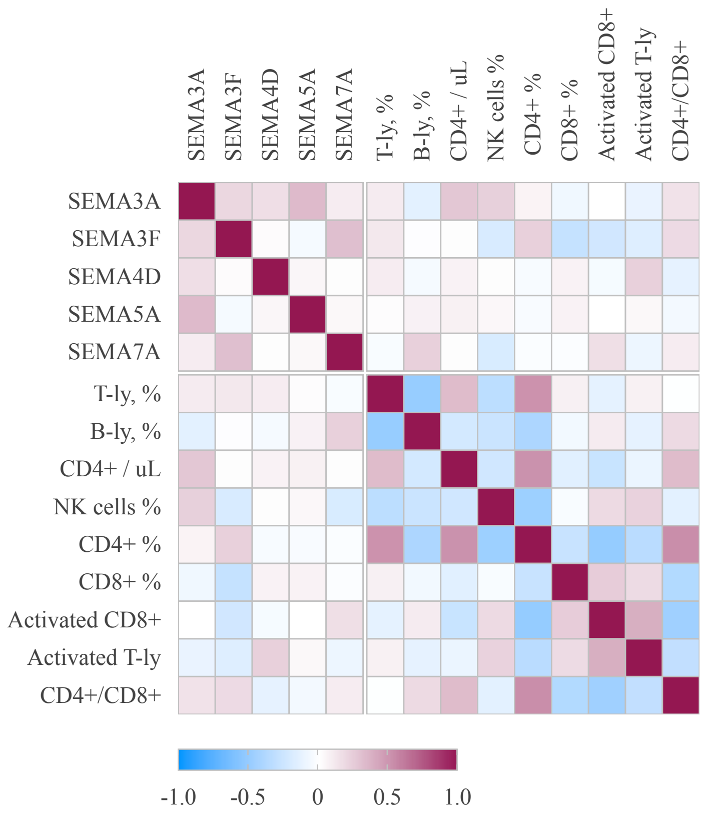

Supplementary Figure S6 – Serum semaphorin and cytokine concentrations at admission (Day 1) and Day 5 in patients with influenza, stratified by MASLD status.

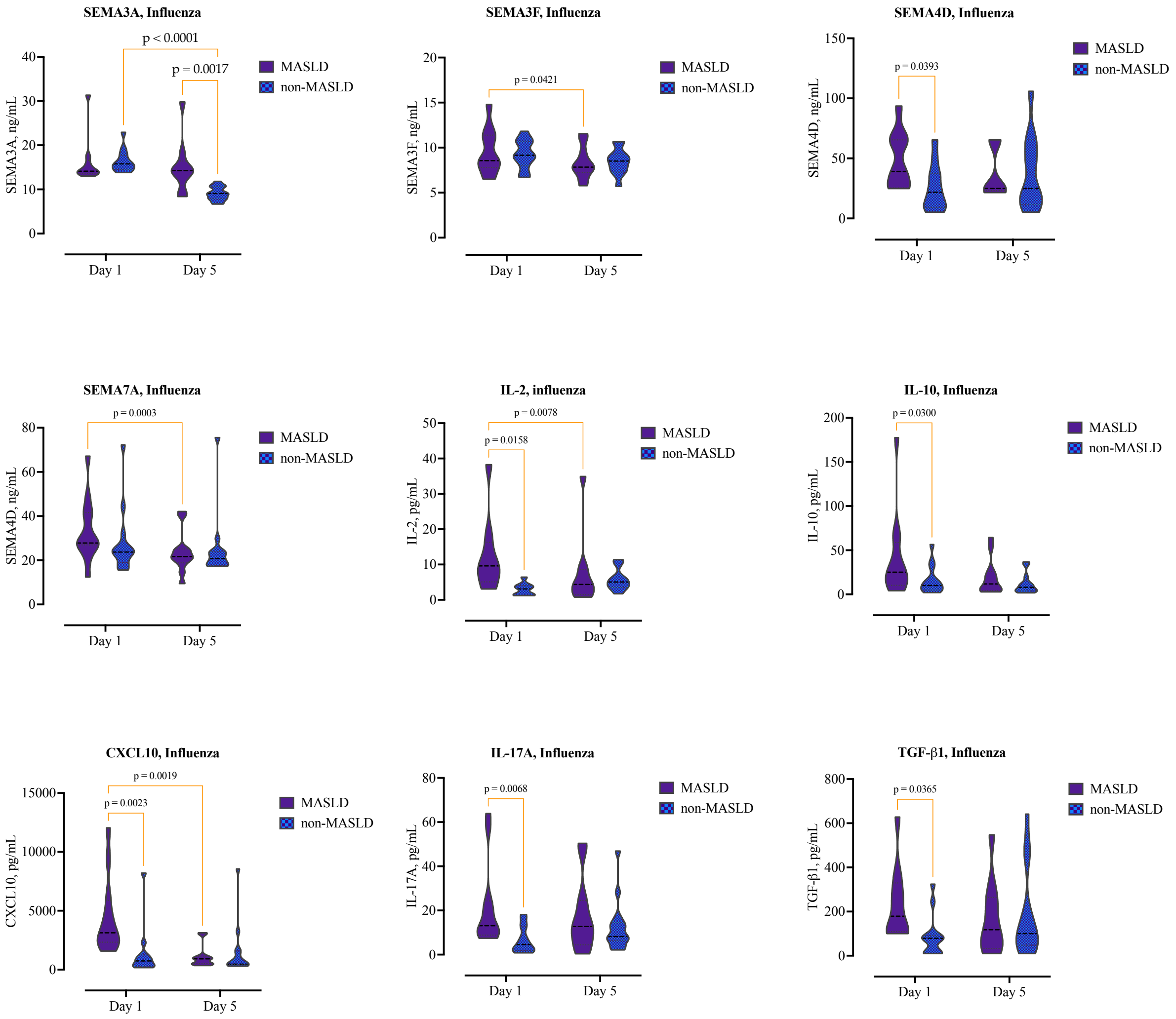

Supplementary Figure S7 – Serum semaphorin and cytokine concentrations at admission (Day 1) and Day 5 in patients with Legionella, stratified by MASLD status.

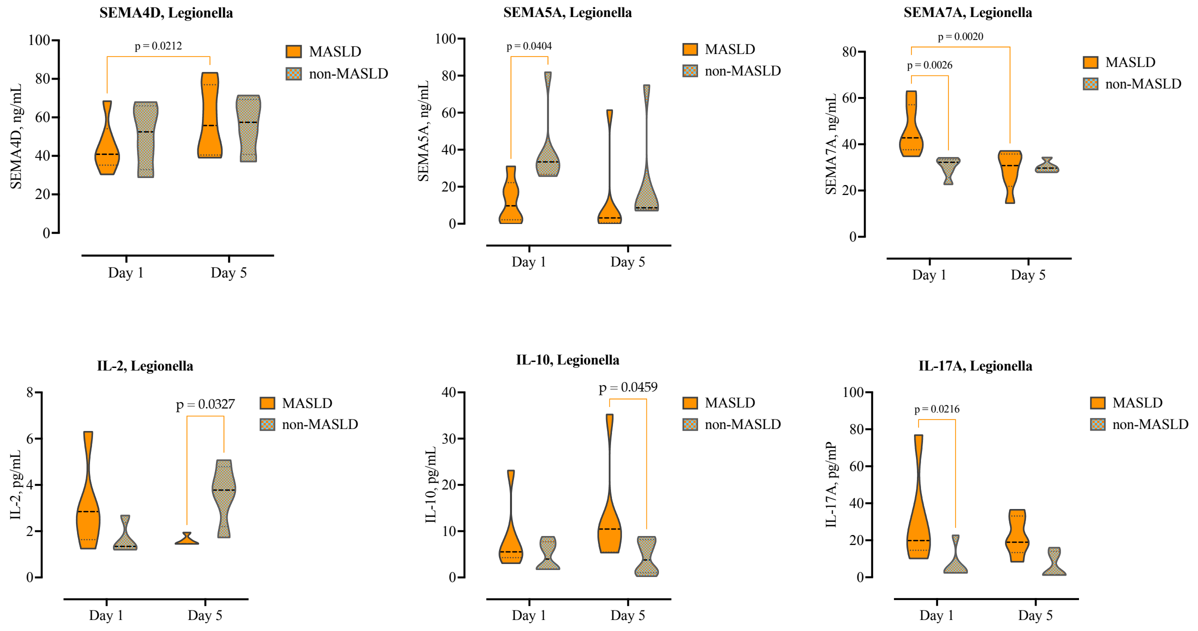

Supplementary Figure S8 – Serum semaphorin and cytokine concentrations at admission (Day 1) and Day 5 in patients with "bacterial pneumonia", stratified by MASLD status.

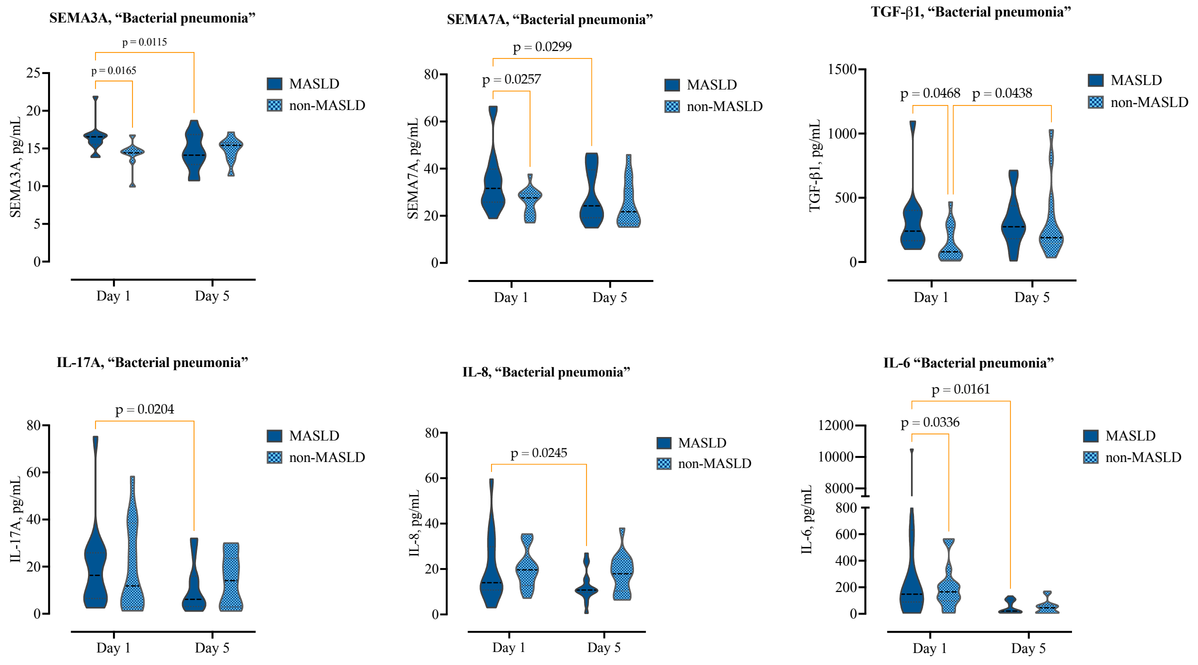

Supplementary Figure S9 – Serum semaphorin and cytokine concentrations at admission (Day 1) and Day 5 in patients requiring IMV or simple O2 therapy.

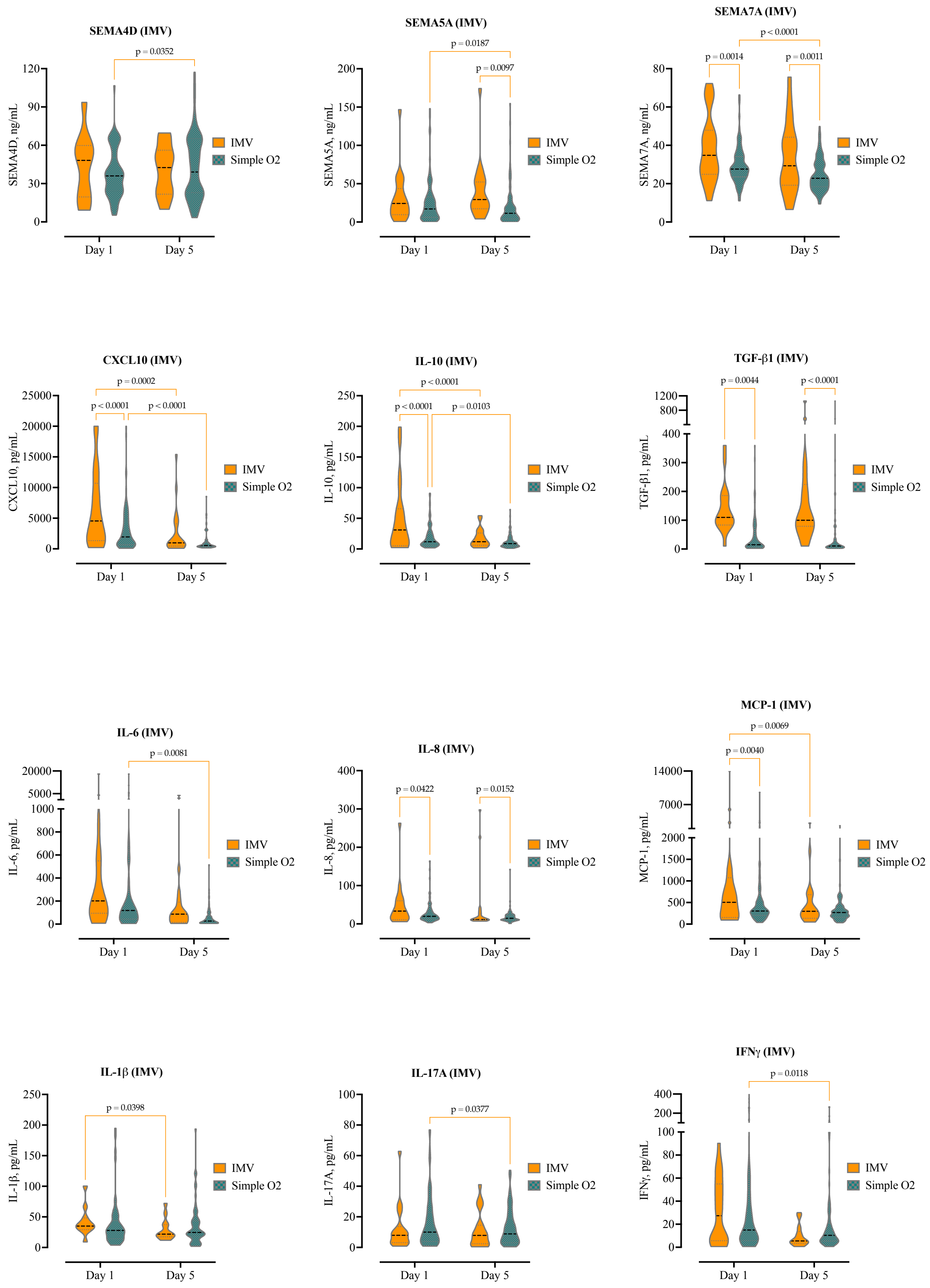

Supplementary Figure S10 – Serum semaphorin and cytokine concentrations at admission (Day 1) and Day 5 in patients requiring CRRT.

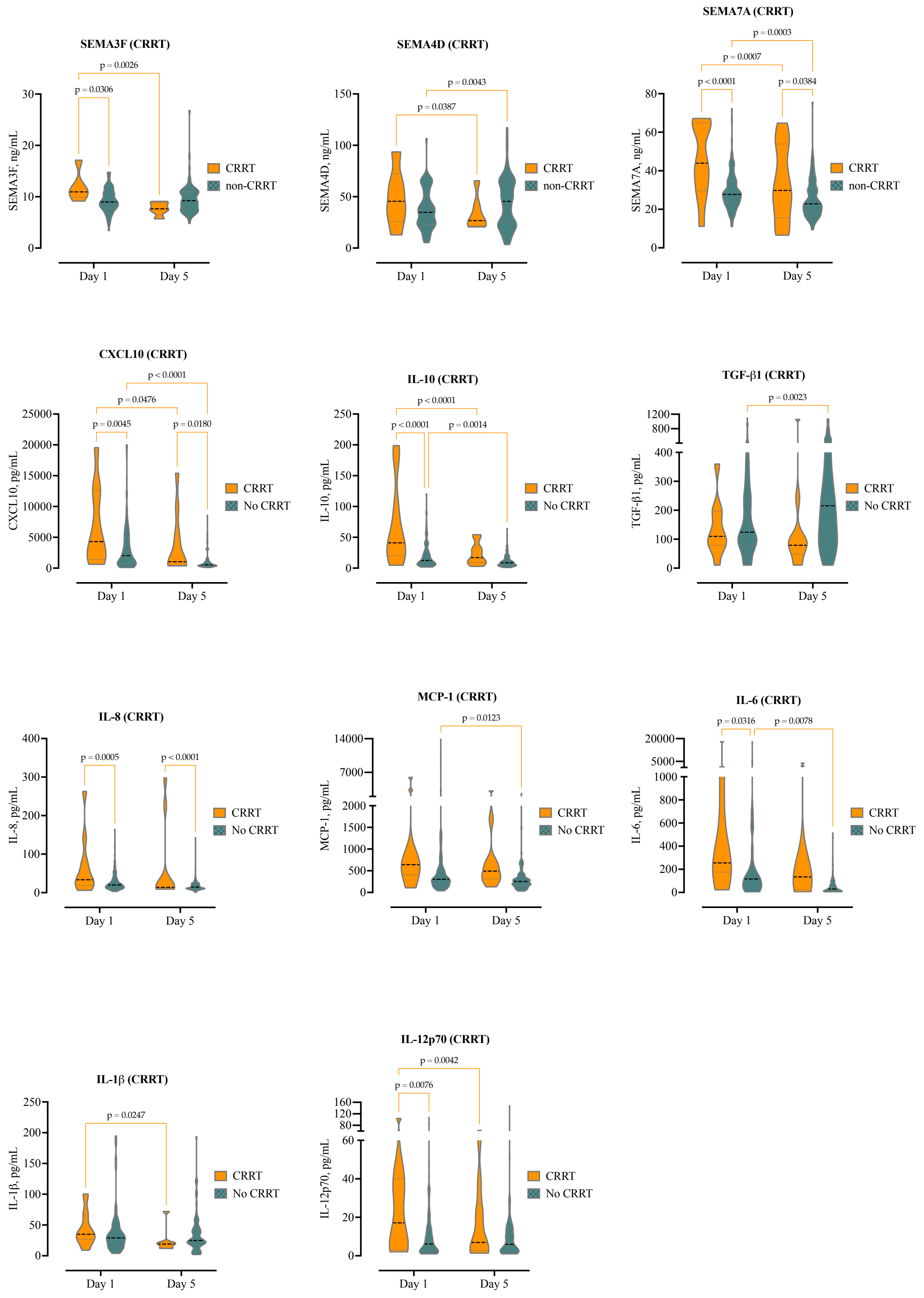

Supplementary Figure S11 – Serum semaphorin and cytokine concentrations at admission (Day 1) and Day 5 in patients with shock.

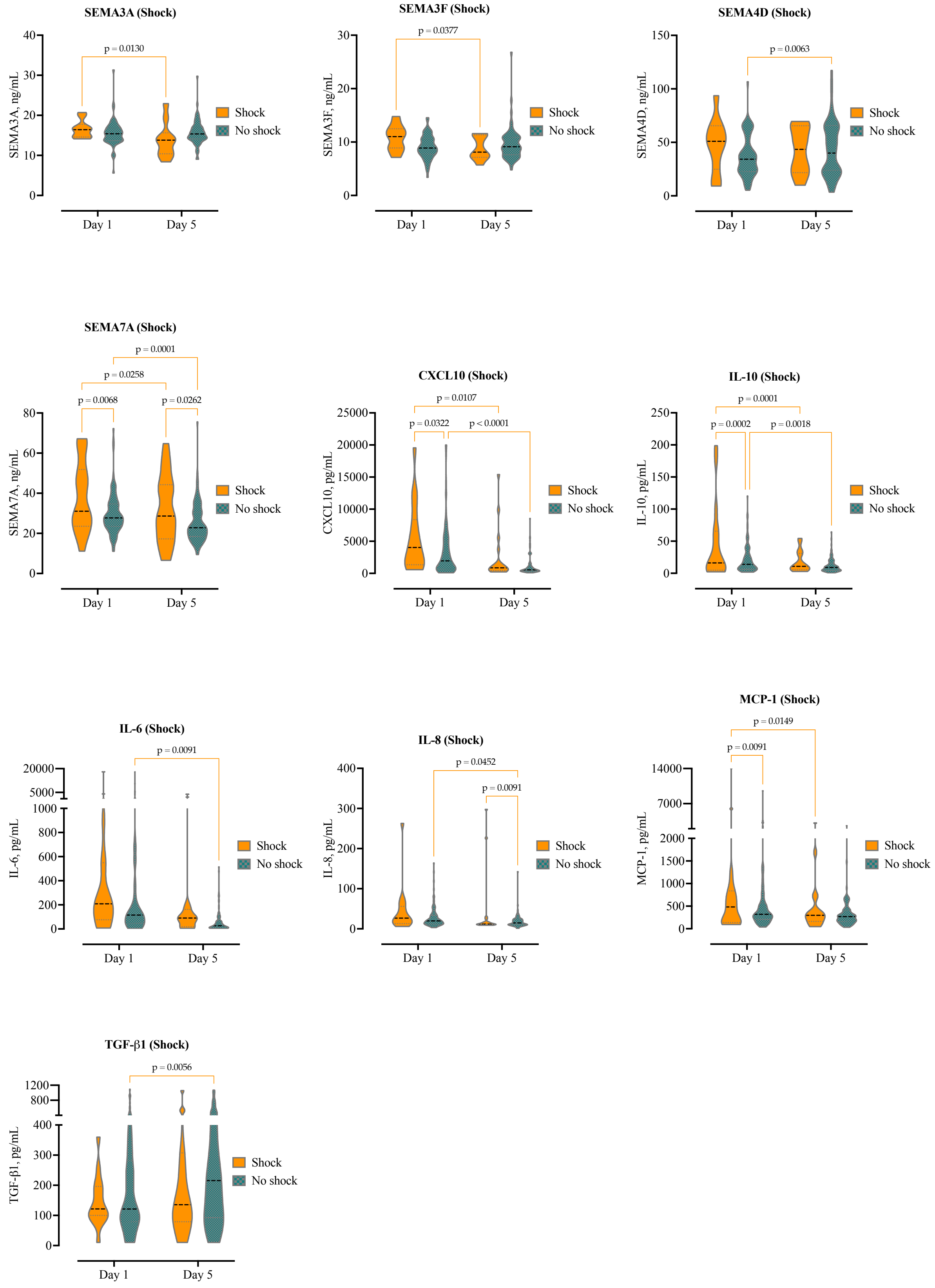

Supplementary Figure S12 – Serum SEMA4D and SEMA7A concentrations at admission (Day 1) and Day 5 comparison between survivals and died patients. Kaplan–Meier survival analysis based on baseline SEMA4D concentrations and ΔSEMA7A (day 1-day 5) concentrations. Log-rank test was used to assess statistical significance.

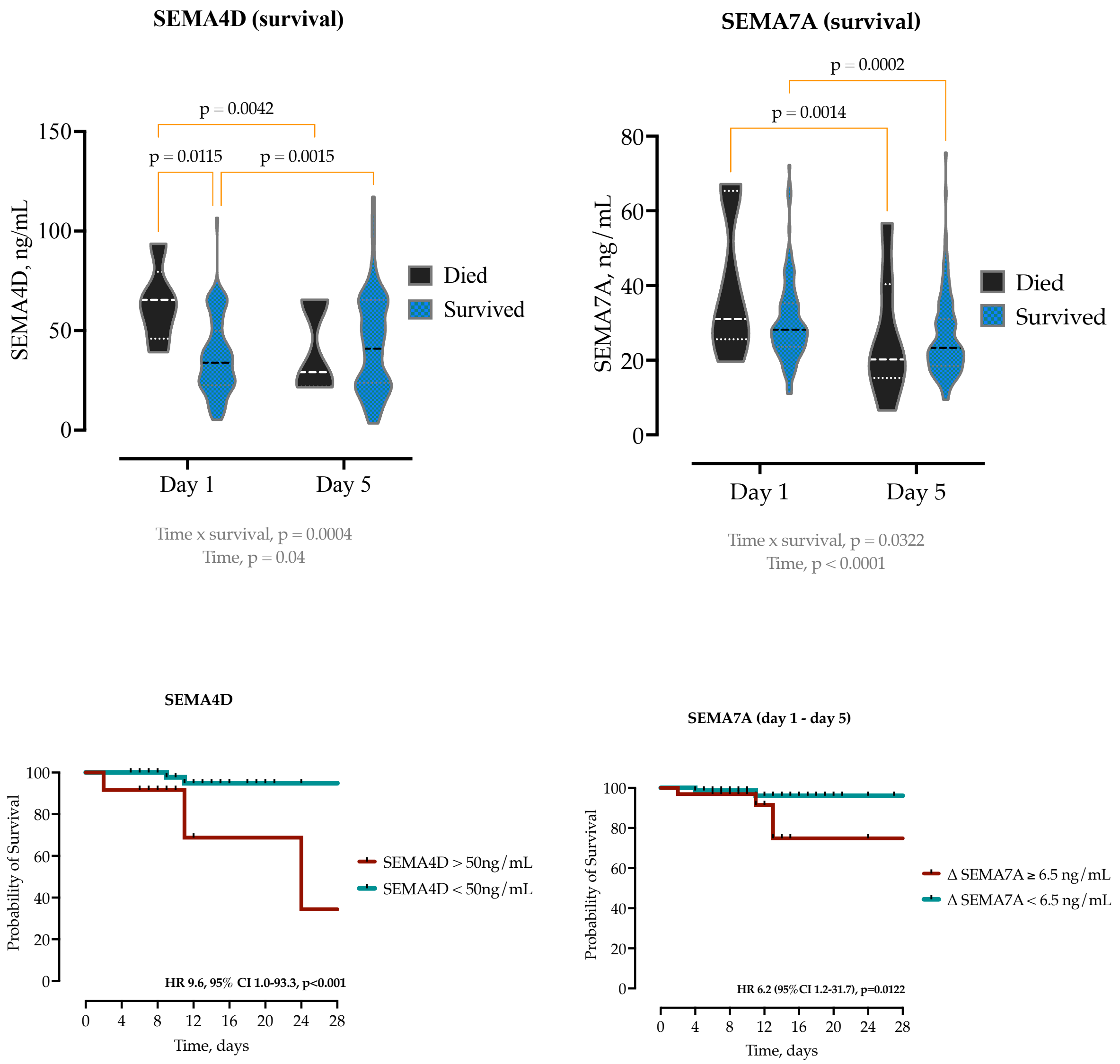

Supplement: Supplementary file 1 [file ijms-26-08095-s001.zip › ijms-3760688-supplementary.pdf]
